# Spatial weed distribution models under climate change: a short review

**DOI:** 10.7717/peerj.15220

**Published:** 2023-04-10

**Authors:** Javier López-Tirado, Jose L. Gonzalez-Andújar

**Affiliations:** 1 Department of Botany, Ecology and Plant Physiology, University of Cordoba, Cordoba, Spain; 2 Department of Crop Protection, Instituto de Agricultura Sostenible, Consejo Superior de Investigaciones Cientificas (CSIC), Cordoba, Spain

**Keywords:** Species Distribution Models (SDMs), Invasive species, Weeds, *Ambrosia artemisiifolia*, *Ageratina adenophora*, *Mikania micrantha*, *Parthenium hysterophorus*

## Abstract

Climate change is a concern worldwide that could trigger many changes with severe consequences. Since human demography is steadily increasing, agriculture has to be constantly investigated to aim at improving its efficiency. Weeds play a key role in this task, especially in the recent past and at present, when new introductions have been favoured by a rise in tourism and international trade. To obtain knowledge relating weeds and their behaviour to climate change, species distribution models (SDMs) have also increased recently. In this work, we have reviewed some articles published since 2017 on modelled weeds, aiming to give a response to, among other things, the species most studied, the scale and location of the studies, the algorithms used and validation parameters, global change scenarios, types of variables, and the sources from which the data were collected. Fifty-nine articles were selected to be reviewed, with maximum entropy (MaxEnt) and area under the curve (AUC) being the most popular software and validation processes. Environmental and topographic variables were considered above pedological and anthropogenic ones. Europe was the continent and China, the USA, and India the countries most studied. In this review, it was found that the number of published articles between developed and developing countries is unbalanced and comes out in favour of the former. The current knowledge on this topic can be considered to be good not enough, especially in developing countries with high population densities. The more knowledge we can obtain, the better our understanding is of how to deal with this issue, which is a worldwide preoccupation.

## Introduction

Given its impact on numerous socioeconomic sectors of human activity, global climate change is one of the primary concerns for the future sustainability of our development. Associated with these potential impacts, there will be major changes in global ecosystems, predicting a reduction of between 10% and 20% of world production in agricultural ones (IPCC, 2007).

The expected impact of climate change on agricultural ecosystems is variable (Newton et al., 2006). Some agricultural regions will be threatened by climate change, while others could benefit from it. For instance, northern Europe could gain from rising temperatures, while southern Europe, where droughts are more likely, may experience longer and more severe dry periods. The climate is also expected to become more variable than it is at present, especially in semi-arid and arid regions, causing fluctuations in crop yields.

The effects of climate change will have a profound influence on crop protection, affecting pests, diseases, and weeds. Especially affected will be the weed flora, involving their physiology and biological cycle, and competitive relationship between weeds and crops (Gonzalez-Andujar, 1995). Climate change may also influence the geographic distribution of existing weeds or the invasion of crops by new plant species. Some studies have documented the displacement of species such as *Amaranthus retroflexus*, *Abutilon theophrasti*, and *Avena sterilis* (Castellanos-Frías et al., 2014; Peters, Breitsameter & Gerowitt, 2014). In the last decades, tourism and international trade (especially by sea) have emerged as important factors favouring plant invasions and promoting the arrival of propagules (Meyerson & Mooney, 2007). Anthropogenic disturbances such as changes in the availability of limiting factors and landscape fragmentation help to facilitate the settlement of invaders once they have arrived (Le Maitre, Richardson & Chapman, 2004). In this sense, it be said that today the world has shrunk, with new invasions being a threat anywhere (Hulme, 2009), so that invasion pathways have to be identified and managed to prevent the introduction of exotic species (Hulme, 2015). The importance of controlling these invasion pathways relapses, especially in live plant displacement (some of the weed species having been introduced as crops) which could be transmitted to animals or pathogens *via* vectors (Liebhold et al., 2012). To combat weed success, it is encouraged to research and model native species used in crops that prevent their invasion as in the case of *Ipomoea batatas* (L.) Lam., and *Vitex negundo* L. in China (Fang et al., 2021). In the same context, parasitic plant species must also be taken into account too, as has been supported by recent studies (Cai et al., 2022).

Crop species can also become weeds, as has happened with transgenic crops in Canada, and so can glyphosate-resistant crops, spreading the weed problem world-wide (Duke & Powles, 2009). Hybridization and genetic changes with other species pose a new problem in the interrelationship between crops and weeds (Warwick, Beckie & Small, 1999).

Predictive models of the geographic distribution of species are powerful tools that can be the assistance in decision-making on the management of weed flora under different climate scenarios. Different types of models have been developed using a wide variety of computer software (*i.e*., CLIMEX, BIOCLIM, maximum entropy, *etc*.). Most of them are based on the concept of a bioclimatic niche and on the use of eco-physiological data for the species, whose objective is to predict the capacity of plant species to invade new habitats under different climatic conditions (Kriticos, Yonow & Mcfadyen, 2005).

In this article, we have carried out a cursory review of weed distribution models since 2017. Algorithms, validation processes, projections at different time slices, variables and sources of extraction, frequency of the studied weeds, and study area are provided and discussed.

## Material and methods

A review of Web of Science (https://www.webofscience.com/) was carried out, considering all the databases and collections. The keywords “weed species distribution models” were inserted in the search as the topic, and a time-slice from 2017 to 2021 was defined. Only the articles that dealt with the following two criteria were taken into account: (i) harmful weeds for crops and (ii) weeds that were modelled by means of species distribution, *i.e*., prediction of suitable areas by niche-based models’ approach.

A database was created, including: (i) information on the references (title, authors, year, *etc*.) and their content; (ii) the number and name of the species studied; (iii) their origin (native or non-native), study area (global, continent, country, or region), algorithm(s) used; (iv) the validation method(s), and whether the species were forecasted and/or hindcasted. The list of 59 reviewed articles is available as Material S1.

Additionally, the above information enabled us to retrieve some statistics on the species most studied and the algorithms and validation methods most commonly used, among others. Characteristics like the extent of the study area and the predictions for periods other than the present were also significant. The species were grouped by families following the International Plant Names InDex (https://www.ipni.org/).

## Results and Discussion

### General output

Global climate change affects human health and wellbeing due to more extreme weather events and wildfires, decreased air quality, and diseases transmitted by insects, food, and water. Climate disruptions to agriculture have been increasing and are projected to become more severe over this century, a trend that would affect pest distributions and their effect on crops. In this regard, it is important to find out the future spatial distribution of weeds in order to predict and mitigate their impact on crops. Since the last few decades, species distribution models (SDMs) have been appearing to answer questions about how living beings could behave in the upcoming years. A huge number of groups of plants, animals, fungi, pathogens, *etc*. are being studied from a conservational and an economic point of view (Bellard et al., 2018; Lantschner, de la Vega & Corley, 2019). According to the latter, weeds are significantly important and must be kept under control in croplands. Many similar studies have been published in the past few years all over the world.

The results yielded were analysed one by one. After applying the criteria for the subject of this review, 59 articles were deemed suitable for a deeper assessment. The number of articles published per year was as follows: 12 in 2017, 7 in 2018, 14 in 2019, 14 in 2020, and 12 in 2021. Forty-two out of 59 articles dealt with only one species, 10 included between two and nine species, and six studied between 11 and 20 species. The remaining reference considered 32 species (Shabani et al., 2020).

Thirty-four out of 59 articles added forecasts in one or more scenarios to the study of the present period. Hindcasting was much rarer and only developed in four of them; only one article used remote periods like the last glacial maximum (~21,000 years BP) and mid-holocene (~6,000 BP), whilst the remaining three articles covered more recent periods during the 20th and the early 21st centuries (Hicks et al., 2021; Mang et al., 2018; Ren et al., 2020; Runquist et al., 2019).

In general terms, the authors of the articles reviewed did not mention the types of crops in which the weeds grew. In a few cases, beet, chaste tree, coconut, eucalyptus, maize, mustard, oats, palm oil, sugarcane, potato, rice, rubber, soybean, sunflower, sweet potato, teak, and wheat crops were mentioned.

Our literature review confirms the concern about weeds and the expected global changes (IPCC, 2007). Hindcasting does not seem to be of great interest to most of the researchers, while forecasting in the next few decades is the issue most studied. The authors of the few articles on hindcasting focused their research on recent periods, and their results could be of interest for studying weed distribution in the last few decades or years. Monitoring their tendency from the past to the future throughout the years and having broader predictions would be useful, but, in our opinion, examining hindcasting from other eras (a thousand years ago) does not make much sense.

### Weeds studied

A total of 130 species were studied 194 times in the 59 articles. It should be noted that some researchers published more than one article on the same species.

The species most often studied was *Ambrosia artemisiifolia* L. (eight times), followed by *Ageratina adenophora* (Spreng.) R.M.King & H.Rob., *Mikania micrantha* Kunth, *Parthenium hysterophorus* L. (six times), and *Imperata cylindrica* (L.) Raeusch. The rest of the species were only studied once and can be consulted in Material S2.

The species studied mostly came either from non-native areas (108 times) or native and non-native areas together (79 times)—the latter, generally on a global scale. Only seven times were the species studied in their natural habitat. This is consistent with the expected predictions on climate change and the consequences it could have on invasive species (Elith, 2017). The most flagrant evidence for studying alien weeds rather than native ones can be attributed, in our view, to the fact that exotic plants could become more aggressive and invasive in the current context of climate change. Globalization could also be involved in this trend, since nowadays tourism and business promote seed exchange worldwide (Song, Li & Cao, 2018).

The families with the species more studied in the reviewed articles were Asteraceae (36 species) and Poaceae (23 species), *i.e*., both families assumed 45% of the total. Families with more than one studied weed in the reviewed articles are shown in Table 1. The remaining 19 families were represented by only one species (see Material S2).

**Table 1:** Botanical families with more than one studied weed in the reviewed articles.

Family	Number of species
Asteraceae	36
Poaceae	23
Fabaceae	6
Asparagaceae	6
Amaranthaceae	5
Brassicaceae	5
Malvaceae	5
Apiaceae	3
Chenopodiaceae	3
Euphorbiaceae	3
Scrophulariaceae	3
Solanaceae	3
Cactaceae	2
Caryophyllaceae	2
Geraniaceae	2
Ranunculaceae	2
Verbenaceae	2

The most common families were Asteraceae, Poaceae, and Fabaceae (Devesa Alcaraz & Carrión García, 2012). Therefore, weeds are also well represented by these families, and they have been extensively investigated in the last few years, according to our results. *Ambrosia artemisiifolia* is an aggressive weed that has been considered from ecophysiological, allergenic, and chemical points of view (Willemsen & Rice, 1972; Bazzaz, 1974; Wang, Xu & Wu, 1993; Klimek, Becker & Raulf, 2014). In fact, according to our review, the Asteraceae species is the one most investigated. Other families like Chenopodiaceae and Amaranthaceae were also of interest, especially the Amaranthus genus. The repetition of some emblematic taxa was due to their concern and interest. For instance, in China, several species were repeatedly studied by Wan & Wang (2019a, 2019b).

### Origin of data: variables and databases

Climatic or bioclimatic variables were always used when setting up models, following the example of Bede-Fazekas & Somodi (2020), who frequently considered them. In some cases, these explanatory variables were used alone, whereas in others, they were combined with topographic, pedological, anthropogenic (human footprint), and, to a lesser extent, other biotic variables.

Topographic variables also supported the climatic or bioclimatic ones, although the authors were generally accurate, and correlation matrices were computed in order to prevent collinearity between variables. Human footprints or anthropogenic variables are of interest in modelling (Seiferling, Proulx & Wirth, 2014; Gallardo, Zieritz & Aldridge, 2015), especially when working with weeds (Korres et al., 2016). Biotic variables have been increasing in the last few years, although they are not used very much yet, and, in this review, very few articles considered them. The target species (weeds) may not show the same suitability as forestry or non-weedy species, where biotic variables can provide a better fit to the niche (Woerner & Hackney, 1997; Meier et al., 2010).

The databases for obtaining the information were selected according to the extent of the study area, and the availability of data per country. In general, big countries and a group of countries or continents were the areas most studied, so global databases were frequently used. Local studies, on the other hand, were drafted compiling data from national databases. This argument is in accordance with the resolution, *i.e*., lower resolution at larger areas and *vice versa*. The WorldClim project (https://www.worldclim.org) was the most extensive database considered, especially when modelling all around the world, bioclimatic variables and altitude. Other topographic variables, like aspect, were computed with GIS from the latter. In some cases, the Shuttle Radar Topographic Mission (SRTM) was also resorted to, and as for the pedology, the most popular work used was SoilGrids (https://soilgrids.org). Human footprint variables were a resource that the authors did not discard and impacts and disturbances were procured primarily from the Socioeconomic Data and Applications Centre (SEDAC), the History Database of the Global Environment (HYDE), and the Range and Watershed Management Organization (FRWMO). The EarthEnv dataset and the Global Land Cover Facility (GLCF) were also taken into consideration for biotic variables. The more local the study area, the more local the sources were.

Occurrence data for dependent variables were normally obtained from the Global Biodiversity Information Facility portal (www.gbif.org) and sometimes enriched with unpublished data by the authors of the reviewed articles.

### Spanning and location of the studies

**Figure 1:**
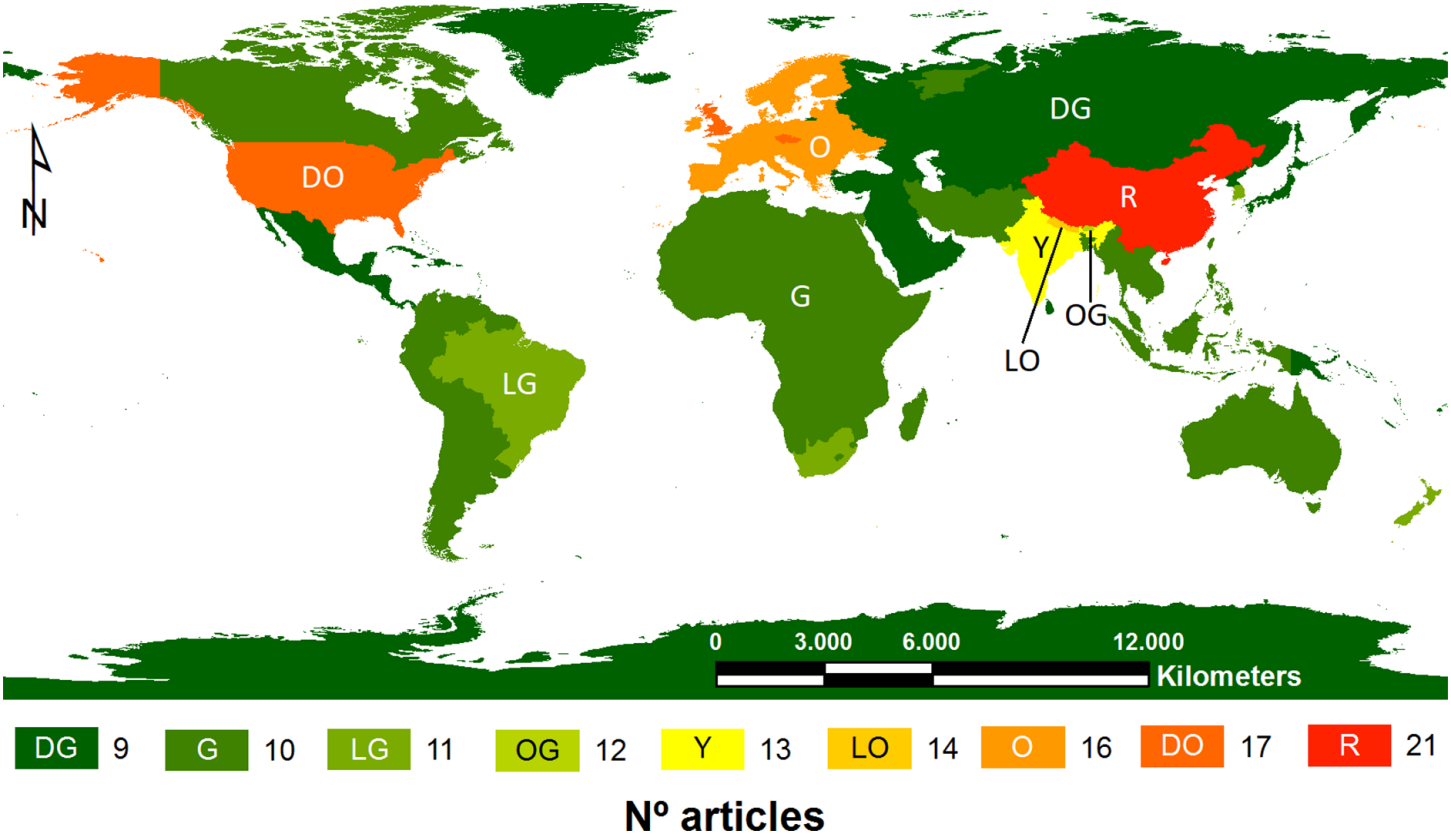
Number of articles reviewed per study area. DG, dark green; G, green; LG, light green; OG, olive green; Y, yellow; LO, light orange; O, orange; DO, dark orange; R, red.

Nine articles were focused on the world, 13 on a continental scale (Two of them considering two different continents), 21 on countries, 10 on regions, and six at local divisions. Specifically, three references belonged to Africa, 11 to America, 24 to Asia, 11 to Europe, and three to Oceania (Fig. 1). Per country, China published the highest number of the reviewed articles, followed by the USA and India. Brazil in South America and South Africa in Africa were also remarkable on their respective continents.

To model our planet implies working with a lower resolution due to data weight and computers’ performance. This is consistent with other scientific research (Guisan et al., 2007; Franklin & Miller, 2010), and a balanced occurrence and explanatory data in each part of the globe are crucial. Resolution changes can influence the perception and behaviour of the models (Lantschner, de la Vega & Corley, 2019). The smaller study area, however, produces higher resolutions and there are likely to be more accurate presence data for the species.

Regarding location, there was a clear difference in the number of published articles between those administrative areas with facilities to obtaining funding and those that did not invest in the same way. Per continent, Europe is noteworthy, whereas per country, China, the USA, and India are at the forefront. On the other hand, Australia, Russia, South America, and Africa are behind (although Brazil and South Africa are faintly highlighted, respectively). In most cases, differences in their investment to fight climate change are notorious between developing and developed countries (Chinowsky et al., 2011). On a smaller scale, Bhutan, Nepal, and South Korea should be mentioned.

### Model algorithm and validation

The most popular algorithm used was Maximum Entropy (MaxEnt) with thirty-seven out of 59 articles exclusively applying this software. In addition, nine studies considered several algorithms; MaxEnt was also present in five of them, so that the latte was employed in 71.2% of the cases. Random forest (RF) followed MaxEnt, although at a considerably greater distance. This algorithm was used in six articles; only in one case was it used alone. By contrast, CLIMEX was found in four articles by itself, whereas once it was used together with other algorithms. Other algorithms like artificial neural network (ANN), classification tree analysis (CTA), generalized boosting model (GBM), generalized linear models (GLM), multiple adaptive regression splines (MARS), logistic regression (LOGIT), *etc*. were used less (Table 2).

**Table 2:** Niche-based modelling algorithm, approach, and number of articles reviewed.

Niche-based modelling algorithm	Modelling approach	N° articles
Maximum Entropy (MaxEnt)	Correlative	42
Generalized Linear Model (GLM)	Correlative/Mechanistic	6
Random Forest (RF)	Correlative	6
Climex	Semi-mechanistic	5
Artificial Neural Network (ANN)	Correlative	4
Generalized Boosting Model (GBM)	Correlative	4
Boosted Regression Trees (BRT)	Correlative	3
Classification Tree Analysis (CTA)	Correlative	3
Generalized Additive Models (GAM)	Correlative	3
Multivariate Adaptive Regression Splines (MARS)	Correlative	2
Surface Range Envelope (SRE)	Correlative	2
Flexible Discriminant Analysis (FDA)	Correlative	1
Forward Mechanistic Distribution Models	Mechanistic	1
Gradient Boosting Machine (GBM)	Correlative	1
Hierarchical Bayesian Modelling	Correlative	1
Life Cycle	Mechanistic	1
Logistic Regression (logit)	Correlative	1
Thornley Transport Resistance (TTR)	Correlative	1

Among the various model validation methods, Area Under the Curve (AUC) stood out significantly. Forty-five articles took it into consideration, of which 28 only considered AUC. The true skill statistic (TSS) was also largely used in the reviewed works: 15 times, of which only once was considered without any other validation method. Besides AUC and TSS, two articles also used a third method called Kappa. Bayesian information criterion (BIC) and root mean squared error (RMSE), among others, were also methods applied.

As can be seen from the results, most of the authors agreed on using MaxEnt and AUC for their respective works, which is consistent with other SDM studies not on weeds. Currently, both MaxEnt and AUC seem to be robust methodologies in this field (Cobben et al., 2015; Cao et al., 2016). In general, AUC gave accurate values according to Swets (1988), who ranked those values as follows: low accuracy (0.5–0.7), potentially useful (0.7–0.9), and high accuracy (>0.9). Wan, Wang & Yu (2019) established 0.7 as a relevant threshold value. The fact that TSS is normally used together with AUC may be due to the weakness of the former. Thus, other alternatives are being investigated to assess SDMs, such as the odds ratio skill score (ORSS) and the Symmetric Extremal Dependence Index (SEDI) (Wunderlich et al., 2019).

MaxEnt is a software programme frequently used worldwide to estimate the potential distribution of invasive plants (Mainali et al., 2015; Merow, Smith & Silander, 2013). This algorithm can perform accurate predictions with few occurrence data (Pearson et al., 2007), although a good management of the dependent and independent or explanatory variables, together with an appropriate election of the resolution, improves MaxEnt performance (Wan, Wang & Yu, 2019). The striking dominance of MaxEnt in plant models contrasts with other groups reviewed, including plants, animals, and pathogens; CLIMEX is commonly used in pest risk assessment (Lantschner, de la Vega & Corley, 2019). However, it has been found that the best statistical SDMs and environmental data substantially outperformed CLIMEX and ecophysiological data when a pest was modelled (Early et al., 2022). The popularity of MaxEnt led to investigating it more profoundly, allowing improvements to be made when managing data and settings. MaxEnt’s strength is that it is easy to use (Mainali et al., 2015; Merow, Smith & Silander, 2013), producing robust outputs when data are scarce, irregularly sampled, or show minor location errors (Elith et al., 2006; Pearson et al., 2007). Moreover, when the study area so allows, spatial filtering can minimize omission errors (false negatives) and commission errors (false positives) (Kramer-Schadt et al., 2013). The number of occurrences, the number of environmental variables, and the spatial scales can also be managed to enhance the performance of MaxEnt. Choosing an appropriate number of occurrences and variables is crucial, whereas using a spatial resolution of 5.0 arc-min to model invasive plants globally seems suitable (Wan, Wang & Yu, 2019).

The use of ensemble models has been encouraged because niche-based modelling algorithms can then be compared, and they can help to understand how their approaches perform on their own. In the current review, ensemble models were used in some cases (nine out of 59 reviewed articles), although only the final consensus maps were provided. In our opinion, this information should not be wasted as it would motivate the authors to publish and discuss their results separately by algorithm.

## Conclusions

The scientific community is responding well to concerns about weeds, agriculture, and climate change. Studies are being carried out around the world, and they are being steadily published (Cai et al., 2022). Nonetheless, there is still a gap to be filled, depending on the weeds and the areas and crops that they colonize. Today, species distribution models (SDM) are being established as a powerful tool to deal not only with weeds, but also with invasive species. We found that the environmental variables, followed by the topographic ones, were those most used when modelling, and that most authors also considered the MaxEnt software and AUC validation process favourably. In any case, the methodology can always be improved, and we hope that the results from this work are an encouragement to continue investigating to obtain the best performance of the models every time.

Other works, such as that of Lantschner, de la Vega & Corley (2019), have reviewed weeds among other organisms, and, in fact, any further reviews of weeds studies would help to improve the knowledge on this subject. Improving this research line would allow us to achieve greater efficiency in producing essential crops for humans, such as rice and wheat, among others.

## Supplemental Information

10.7717/peerj.15220/supp-1 Supplemental Information 1 Reviewed articles.

10.7717/peerj.15220/supp-2 Supplemental Information 2 Species (and their respective families) only studied one time in the revised manuscripts.

## Additional Information and Declarations

### Data Availability

The following information was supplied regarding data availability: This is a literature review.
