# Supplementary material for: Spatial weed distribution models under climate change: a short review
Source: PeerJ. 2023 Apr 10;11:e15220. doi: 10.7717/peerj.15220 (PMC10100825; doi:10.7717/peerj.15220)
Supplement: Supplemental Information 1 [file peerj-11-15220-s001.docx]

**Supplementary Material 1**

Reviewed articles

Ahmad R, Khuroo AA, Hamid M, Charles B, Rashid I (2019) Predicting invasion potential and niche dynamics of *Parthenium hysterophorus* (Congress grass) in India under projected climate change. Biodivers Conserv 28:2319-2344

Alaniz AJ, Nunez-Hidalgo I, Carvajal MA, Alvarenga TM, Gomez-Cantillana P, Vergara PM (2020) Current and future spatial assessment of biological control as a mechanism to reduce economic losses and carbon emissions: the case of *Solanum sisymbriifolium* in Africa. Pest Manage Sci 76:2395-2405

Almasieh K, Zoratipour A, Negaresh K, Delfan-Hasanzadeh K (2018) Habitat quality modelling and effect of climate change on the distribution of *Centaurea pabotii* in Iran. Span J Agric Res 16:e0304

Arogoundade AM, Odindi J, Mutanga O (2020) Modelling *Parthenium hysterophorus* invasion in KwaZulu-Natal province using remotely sensed data and environmental variables. Geocarto Int 35:1450-1465

Baidar T, Shrestha AB, Ranjit R, Adhikari R, Ghimire S, Shrestha N (2017) Impact assessment of *Mikania micrantha* on land cover and MaxEnt modeling to predict its potential invasion sites. In: International Archives of the Photogrammetry Remote Sensing and Spatial Information Sciences 2017 ISPRS Hannover Workshop / High-Resolution Earth Imaging for Geospatial Information (HRIGI) Workshop / City Models, Roads and Traffic (CMRT) Workshop / Image Sequence Analysis (ISA) Workshop / European Calibration and Orientation (EuroCOW) Workshop, Hannover, Germany:305-310

Banerjee AK, Mukherjee A, Dewanji A (2017) Potential distribution of *Mikania micrantha* Kunth in India - evidence of climatic niche and biome shifts. Flora 234:215-223

Banerjee AK, Mukherjee A, Guo W, Ng WL, Huang Y (2019) Combining ecological niche modeling with genetic lineage information to predict potential distribution of *Mikania micrantha* Kunth in South and Southeast Asia under predicted climate change. Global Ecol Conserv 20:e00800.

Bateman TM, Villalba JJ, Ramsey RD, Sant ED (2020) A Multi-Scale Approach to Predict the Fractional Cover of Medusahead (*Taeniatherum Caput-Medusae*). Rangeland Ecol Manage 73:538-546

Bourdot GW, Lamoureaux SL (2021) *Abutilon theophrasti*-a comparison of two climate niche models. N Z J Agric Res 64:211-222

Case MJ, Stinson KA (2018) Climate change impacts on the distribution of the allergenic plant, common ragweed (*Ambrosia artemisiifolia*) in the eastern United States. PLoS ONE 13:e0205677

Certner M, Kur P, Kolar F, Suda J (2019) Climatic conditions and human activities shape diploid-tetraploid coexistence at different spatial scales in the common weed *Tripleurospermum inodorum* (Asteraceae). J Biogeogr 46:1355-1366

Chadin I, Dalke I, Zakhozhiy I, Malyshev R, Madi E, Kuzivanova O, Kirillov D, Elsakov V (2017) Distribution of the invasive plant species *Heracleum sosnowskyi* Manden. in the Komi Republic (Russia). PhytoKeys:71-80.

Changjun G, Yanli T, Linshan L, Bo w, Yili Z, Haibin Y, Xilong W, Zhuoga Y, Binghua Z, Bohao C (2021) Predicting the potential global distribution of *Ageratina adenophora* under current and future climate change scenarios. Ecol Evol 11:12092-12113

Chapman DS, Scalone R, Stefanic E, Bullock JM (2017) Mechanistic species distribution modeling reveals a niche shift during invasion. Ecology 98:1671-1680

Chhogyel N, Kumar L, Bajgai Y (2021) Invasion status and impacts of parthenium weed (*Parthenium hysterophorus*) in West-Central region of Bhutan. Biol Invasions 23:2763-2779

Chikuruwo C, Masocha M, Murwira A, Ndaimani H (2017) Predicting the suitable habitat of the invasive *Xanthium strumarium* L. in southeastern Zimbabwe. Appl Ecol Environ Res 15:17-32

Da Re D, Tordoni E, De Pascalis F, Negrín-Pérez Z, Fernández-Palacios JM, Arévalo JR, Rocchini D, Medina FM, Otto R, Arlé E, Bacaro G (2020) Invasive fountain grass (*Pennisetum setaceum* (Forssk.) Chiov.) increases its potential area of distribution in Tenerife island under future climatic scenarios. Plant Ecolog 221:867-882

Dana ED, Verloove F, Alves P, Heiden G (2021) *Senecio brasiliensis* (Spreng.) Less. (Asteraceae), another potentially invasive alien species in Europe. Bioinvasions Rec 10:521-536

Fang Y, Zhang X, Wei H et al. (2021) Predicting the invasive trend of exotic plants in China based on the ensemble model under climate change: A case for three invasive plants of Asteraceae. Sci Total Environ 756:143841

Hak JC, Comer PJ (2020) Modeling Invasive Annual Grass Abundance in the Cold Desert Ecoregions of the Interior Western United States. Rangeland Ecol Manage 73:171-180

Hannah L, Aguilar G, Blanchon D (2019) Spatial Distribution of the Mexican Daisy, Erigeron karvinskianus, in New Zealand under Climate Change. Climate 7:24

Herrando-Moraira S, Vitales D, Nualart N et al. (2020) Global distribution patterns and niche modelling of the invasive *Kalanchoe x houghtonii* (Crassulaceae). Sci Rep 10:3143

Hicks H, Lambert J, Pywell R et al. (2021) Characterizing the environmental drivers of the abundance and distribution of *Alopecurus myosuroides* on a national scale. Pest Manage Sci 77:2726-2736

Hong SH, Lee YH, Lee G, Lee DH, Adhikari P (2021) Predicting Impacts of Climate Change on Northward Range Expansion of Invasive Weeds in South Korea. Plants 10:1604

Iannella M, De Simone W, D'alessandro P, Console G, Biondi M (2019) Investigating the Current and Future Co-Occurrence of *Ambrosia artemisiifolia* and *Ophraella communa* in Europe through Ecological Modelling and Remote Sensing Data Analysis. Int J Environ Res Public Health 16:3416

Kistner EJ, Hatfield JL (2018) Potential Geographic Distribution of Palmer Amaranth under Current and Future Climates. Agric Environ Lett 3:170044

Kriticos DJ, Ireland KB, Morin L et al. (2021) Integrating ecoclimatic niche modelling methods into classical biological control programmes. Biol Control 160:104667

Liu D, Wang R, Gordon DR, Sun X, Chen L, Wang Y (2017) Predicting Plant Invasions Following China's Water Diversion Project. Environ Sci Technol 51:1450-1457

Magarey R, Newton L, Hong SC et al. (2018) Comparison of four modeling tools for the prediction of potential distribution for non-indigenous weeds in the United States. Biol Invasions 20:679-694

Maharjan S, Shrestha BB, Joshi MD et al. (2019) Predicting suitable habitat of an invasive weed *Parthenium hysterophorus* under future climate scenarios in Chitwan Annapurna Landscape, Nepal. J Mountain Sci 16:2243-2256

Mang T, Essl F, Moser D, Dullinger S (2018) Climate warming drives invasion history of *Ambrosia artemisiifolia* in central Europe. Preslia 90:59-81

Mccartney KR, Kumar S, Sing SE, Ward SM (2019) Using invaded-range species distribution modeling to estimate the potential distribution of *Linaria* species and their hybrids in the US northern Rockies. Invasive Plant Sci Manage 12:97-111

Mcmahon DE, Urza AK, Brown JL, Phelan C, Chambers JC (2021) Modelling species distributions and environmental suitability highlights risk of plant invasions in western United States. Divers Distrib 27:710-728

Pagnoncelli Jr FDB, Trezzi MM, Gonzalez-Andujar JL (2020) Modeling the Population Dynamics and Management of Italian Ryegrass under Two Climatic Scenarios in Brazil. Plants 9:325

Park JS, Choi D, Kim Y (2020) Potential Distribution of Goldenrod (*Solidago altissima* L.) during Climate Change in South Korea. Sustainability 12:6710

Poudel AS, Shrestha BB, Joshi MD, Muniappan R, Adiga A, Venkatramanan S, Jha PK (2020) Predicting the Current and Future Distribution of the Invasive Weed Ageratina adenophora in the Chitwan-Annapurna Landscape, Nepal. Mt Res Dev 40:R61-R71

Qin Z, Zhang JE, Jiang YP, Wei H, Wang FG, Lu XN (2018) Invasion process and potential spread of *Amaranthus retroflexus* in China. Weed Res 58:57-67

Rasmussen K, Thyrring J, Muscarella R, Borchsenius F (2017) Climate-change-induced range shifts of three allergenic ragweeds (*Ambrosia* L.) in Europe and their potential impact on human health. PeerJ 5:e3104

Ray D, Behera MD, Jacob J (2019) Comparing invasiveness of native and non-native species under changing climate in North-East India: ecological niche modelling with plant types differing in biogeographic origin. Environ Monit Assess 191:793

Ren Z, Zagortchev L, Ma J, Yan M, Li J (2020) Predicting the potential distribution of the parasitic *Cuscuta chinensis* under global warming. BMC Ecol 20:28

Roman JFC, Hernandez-Lambrano RE, Rodriguez De La Cruz D, Sanchez Agudo JA (2021) Analysis of the Adaptative Strategy of *Cirsium vulgare* (Savi) Ten. in the Colonization of New Territories. Sustainability 13:2384

Runquist RDB, Lake T, Tiffin P, Moeller DA (2019) Species distribution models throughout the invasion history of Palmer amaranth predict regions at risk of future invasion and reveal challenges with modeling rapidly shifting geographic ranges. Sci Rep 9:2426

Shabani F, Ahmadi M, Kumar L, Solhjouy-fard S, Shafapour Tehrany M, Shabani F, Kalantar B, Esmaeili A (2020) Invasive weed species' threats to global biodiversity: Future scenarios of changes in the number of invasive species in a changing climate. Ecol Indic 116:106436

Shrestha BB, Pokhrel K, Paudel N, Poudel S, Shabbir A, Adkins SW (2019) Distribution of *Parthenium hysterophorus* and one of its biological control agents (Coleoptera: *Zygogramma bicolorata*) in Nepal. Weed Res 59:467-478

Skalova H, Guo WY, Wild J, Pysek P (2017) *Ambrosia artemisiifolia* in the Czech Republic: history of invasion, current distribution and prediction of future spread. Preslia 89:1-16

Sosa AJ, Cardo MV, Julien MH (2017) Predicting weed distribution at the regional scale in the native range: environmental determinants and biocontrol implications of *Phyla nodiflora* (Verbenaceae). Weed Res 57:193-203

Sun Y, Broennimann O, Roderick GK, Poltavsky A, Lommen STE, Mueller-Schaerer H (2017) Climatic suitability ranking of biological control candidates: a biogeographic approach for ragweed management in Europe. Ecosphere 8:e01731

Thiney U, Banterng P, Gonkhamdee S, Katawatin R (2019) Distributions of Alien Invasive Weeds under Climate Change Scenarios in Mountainous Bhutan. Agronomy 9:442

Tu W, Xiong Q, Qiu X, Zhang Y (2021) Dynamics of invasive alien plant species in China under climate change scenarios. Ecol Indic 129:107919

Wan JZ, Wang CJ (2019a) Determining key monitoring areas for the 10 most important weed species under a changing climate. Sci Total Environ 683:568-577

Wan JZ, Wang CJ, Tan JF, Yu FH (2017) Climatic niche divergence and habitat suitability of eight alien invasive weeds in China under climate change. Ecol Evol 7:1541-1552

Wan JZ, Wang CJ (2019b) Contribution of environmental factors toward distribution of ten most dangerous weed species globally. Appl Ecol Environ Res 17:14835-14846

Wang C, Lin H, Feng Q, Jin C, Cao A, He L (2017) A New Strategy for the Prevention and Control of *Eupatorium adenophorum* under Climate Change in China. Sustainability 9:2037

Wang CJ, Wan JZ (2020) Assessing the habitat suitability of 10 serious weed species in global croplands. Global Ecol Conserv 23:e01142

Wang CJ, Yin GJ, Song ZM, Wan JZ (2019) Evaluating the spread of 10 invasive weeds in Chinese nature reserves under climate change scenarios in consideration of different scales. Appl Ecol Environ Res 17:3513-3533

Xing F, An R, Wang B, Miao J, Jiang T, Huang X, Hu Y (2021) Mapping the occurrence and spatial distribution of noxious weed species with multisource data in degraded grasslands in the Three-River Headwaters Region, China. Sci Total Environ 801:149714

Yan H, Feng L, Zhao Y, Feng L, Wu D, Zhu C (2020a) Prediction of the spatial distribution of *Alternanthera philoxeroides* in China based on ArcGIS and MaxEnt. Global Ecol Conserv 21:e00856

Yan H, Feng L, Zhao Y, Feng L, Zhu C, Qu Y, Wang H (2020b) Predicting the potential distribution of an invasive species, *Erigeron canadensis* L., in China with a maximum entropy model. Global Ecol Conserv 21:e00822

Yang YB, Liu G, Shi X, Zhang WG, Cai XW, Ren ZL, Yao NN, Zhu ZH, Nie H (2018) Where will Invasive Plants Colonize in Response to Climate Change: Predicting the Invasion of *Galinsoga quadriradiata* in China. Int J Environ Res 12:929-938
