# Supplementary material for: Spatial weed distribution models under climate change: a short review
Source: PeerJ. 2023 Apr 10;11:e15220. doi: 10.7717/peerj.15220 (PMC10100825; doi:10.7717/peerj.15220)
Supplement: Supplemental Information 2 [file peerj-11-15220-s002.docx]

**Supplementary Material 2**

Species (and their respective families) only studied one time in the revised manuscripts:

| **Species** | **Family** |
| --- | --- |
| *Abutilon theophrasti* | Malvaceae |
| *Acacia mearnsii* | Mimosaceae |
| *Acanthospermum australe* | Asteraceae |
| *Acer palmatum* | Aceraceae |
| *Aconitum pendulum* | Ranunculaceae |
| *Ajania tenuifolia* | Asteraceae |
| *Allium vineale* | Alliaceae |
| *Alopecurus myosuroides* | Poaceae |
| *Amaranthus viridis* | Amaranthaceae |
| *Ambrosia psilostachya* | Asteraceae |
| *Ambrosia trifida* | Asteraceae |
| *Andropogon gayanus* | Poaceae |
| *Annona glabra* | Annonaceae |
| *Anredera cordifolia* | Basellaceae |
| *Antirrhinum majus* | Scrophulariaceae |
| *Apium leptophyllum* | Apiaceae |
| *Asparagus aethiopicus* | Asparagaceae |
| *Asparagus africanus* | Asparagaceae |
| *Asparagus asparagoides* | Asparagaceae |
| *Asparagus declinatus* | Asparagaceae |
| *Asparagus plumosus* | Asparagaceae |
| *Asparagus scandens* | Asparagaceae |
| *Aster altaicus* | Asteraceae |
| *Astragalus sinicus* | Fabaceae |
| *Austrocylindropuntia* spp. | Cactaceae |
| *Avena fatua* | Poaceae |
| *Bromus japonicus* | Poaceae |
| *Bromus rubens* | Poaceae |
| *Bromus unioloides* | Poaceae |
| *Cassia mimosoides* | Caesalpiniaceae |
| *Casuarina equisetifolia* | Casuarinaceae |
| *Centaurea pabotii* | Asteraceae |
| *Ceratocephala testiculata* | Ranunculaceae |
| *Chenopodium ambrosioides* | Chenopodiaceae |
| *Chromolaena odorata* | Asteraceae |
| *Chrysanthemoides monilifera* | Asteraceae |
| *Cirsium vulgare* | Asteraceae |
| *Coronopus didymus* | Brassicaceae |
| *Cryptostegia grandiflora* | Asclepiadaceae |
| *Cuscuta chinensis* | Cuscutaceae |
| *Daucus carota* | Apiaceae |
| *Dittrichia viscosa* | Asteraceae |
| *Draba verna* | Brassicaceae |
| *Erigeron karvinskianus* | Asteraceae |
| *Erodium cicutarium* | Geraniaceae |
| *Eupatorium odoratum* | Asteraceae |
| *Euphorbia fischeriana* | Euphorbiaceae |
| *Euphorbia hirta* | Euphorbiaceae |
| *Galinsoga parviflora* | Asteraceae |
| *Galinsoga quadriradiata* | Asteraceae |
| *Gnaphalium calviceps* | Asteraceae |
| *Gymnocoronis spilanthoides* | Asteraceae |
| *Halogeton glomeratus* | Chenopodiaceae |
| *Heracleum sosnowskyi* | Apiaceae |
| *Hevea brasiliensis* | Euphorbiaceae |
| *Hieracium aurantiacum* | Asteraceae |
| *Kalanchoe × houghtonii* | Crassulaceae |
| *Koelreuteria elegans* | Apocynaceae |
| *Lactuca serriola* | Asteraceae |
| *Leontopodium leontopodioides* | Asteraceae |
| *Lepidium perfoliatum* | Brassicaceae |
| *Linaria dalmatica* | Scrophulariaceae |
| *Linaria vulgaris* | Scrophulariaceae |
| *Lycium ferocissimum* | Solanaceae |
| *Malva parviflora* | Malvaceae |
| *Medicago sativa* | Fabaceae |
| *Merremia peltata* | Convolvulaceae |
| *Modiola caroliniana* | Malvaceae |
| *Nassella neesiana* | Poaceae |
| *Nassella trichotoma* | Poaceae |
| *Oenothera laciniata* | Onagraceae |
| *Oxytropis ochrocephala* | Fabaceae |
| *Paspalum dilatatum* | Poaceae |
| *Pelargonium alchemilloides* | Geraniaceae |
| *Pennisetum setaceum* | Poaceae |
| *Pereskia aculeata* | Cactaceae |
| *Phyla nodiflora* | Verbenaceae |
| *Piptochaetium montevidense* | Poaceae |
| *Pistia stratiotes* | Araceae |
| *Poa bulbosa* | Poaceae |
| *Potentilla chinensis* | Rosaceae |
| *Praxelis clematidea* | Asteraceae |
| *Pueraria montana* | Fabaceae |
| *Salsola tragus* | Chenopodiaceae |
| *Schismus barbatus* | Poaceae |
| *Senecio brasiliensis* | Asteraceae |
| *Senecio madagascariensis* | Asteraceae |
| *Sida rhombifolia* | Malvaceae |
| *Silene gallica* | Caryophyllaceae |
| *Sisymbrium altissimum* | Brassicaceae |
| *Sisymbrium officinale* | Brassicaceae |
| *Sisyrinchium angustifolium* | Iridaceae |
| *Solanum sisymbriifolium* | Solanaceae |
| *Solidago altissima* | Asteraceae |
| *Spergularia rubra* | Caryophyllaceae |
| *Sphagneticola trilobata* | Asteraceae |
| *Tipuana tipu* | Fabaceae |
| *Tragopogon dubius* | Asteraceae |
| *Tripleurospermum inodorum* | Asteraceae |
| *Urena lobata* | Malvaceae |
| *Vicia sativa* | Fabaceae |
| *Xanthium strumarium* | Asteraceae |
